# Editorial

**Published:** 1865-12

**Authors:** 


### Editorial.

---

Wm. T. G. Morton, and his Extraordinary Pretensions. —Some two or three weeks since, this gentleman appeared in our city, with letters of introduction to many members of the medical faculty of the city, and was kindly received. He visited the Mercy Hospital, he asked for and obtained the privilege of lecturing an hour to the class in the Chicago Medical College, during which he gave some familiar instructions in regard to the mode of inhaling ether, claimed for it entire safety and superiority over all other anæsthetics, and closed with a pretended *history* of the discovery and application of anæsthesia to the relief of pain during surgical operations. In the historical part of his discourse, the omissions and perversions of facts were certainly remarkable. His sole aim seemed to be to impress his audience with the fact that Dr. W. T. G. MORTON was not only the first to discover and apply anæsthesia, but to his unremitting exertions and enormous pecuniary sacrifices, made in overcoming the opposition and prejudices of the profession, the world is at this time indebted for all the blessings derived from the use of anæsthetics. Whether he obtained an audience in the Rush Medical College or not, we do not know.

Subsequently he obtained an opportunity to address the Chicago Medical Society, at its regular Friday evening meeting. There he pursued the same course, so far as relates to the history of anæsthesia, wholly omitting some important facts, distorting others, magnifying molehills of opposition into mountains, and whatever else would tend to convey the impression that to Dr. MORTON, alone, is the world indebted for all the blessings of anæsthetics in surgery, obstetrics, and dentistry. He went back to the most remote and imperfect attempts to alleviate the pain of surgical operations by the use of opiates, inebriants, &c., not forgetting to mention the well-known remark of Sir HUMPHREY DAVY in relation to the use of nitrous oxide gas, but not a word escaped his lips by which the most attentive listener would have supposed that either HORACE WELLS or C. T. JACKSON ever lived. Now, what is the moral difference between the deliberate suppression of well-established facts in relation to any subject under public discussion, and the direct assertion of falsehoods?

But the historical part of his discourse constituted only its preface. The theme on which he dilated with the greatest enthusiasm was, the stubborn opposition, the bitter prejudices, and the unjust assaults he had to encounter from the medical profession, in his efforts to bestow upon it and the world the inestimable blessings of his great discovery, and the consequent anxiety, loss of time, destruction of business, and expenditure of money which he was compelled to bear, in perfecting, introducing, and defending it. Of course, he did not omit to mention, as parallel cases, the honored names of HARVEY and JENNER. To add to the force of his own representations, he had loaded the table of the society with a profusion of pamphlets entitled "Proceedings in behalf of the Morton Testimonial," in which, among many other things, it is asserted over the signatures of two *millionaires* of Boston, "that Dr. MORTON has sacrificed *all his property*, and *all the profits of his profession, in introducing his discovery*, and in establishing his claim, and that he has seriously impaired his health; that he has failed to obtain compensation from the Government for the use of ether in the army and navy, and that he has no hope of any public compensation."

Finally, the conclusion of the lecture brought the pith of the whole matter, namely:—that inasmuch as he had actually sacrificed time, rest, business, money, everything of a material nature, in conferring his great boon upon the world, and after years of patient application both to the legislative and executive departments of the Government, had utterly failed to obtain any remuneration, he was now compelled to appeal from the Government to the people. He therefore clearly indicated to the society his wish that *all* its members would sign an appeal to the wealthy and benevolent citizens of Chicago to make contributions to the "Morton Testimonial," and that a committee be appointed to call a public meeting of citizens, before which Dr. MORTON should present his claims in person. To prevent any unnecessary loss of time, he very kindly and *modestly* laid upon the table an *appeal* already prepared, printed, and pasted upon a blank sheet of paper, in due form for the signa ures. To encourage the timid and give faith to the doubting, the flattering idea was thrown out, that their *autographs* would be so much cherished by him in future, while the medal and decorations of honor received from the Governments of France and Russia came *un*-ostentatiously into view. Of course, this printed appeal was carefully worded in such a manner as to make it a full endorsement of all Dr. MORTON'S claims, and would have made a capital addition to the next edition of the pamphlet of "Proceedings in behalf of the Morton Testimonial."

Several members of the society signed that paper, and the committee asked for was appointed. More than two weeks have since elapsed, but we have heard nothing of the proposed public meeting. Without wasting a word on the brazen-faced impudence of all this, let us turn directly to the three important questions involved in Dr. MORTON'S pretensions:—

1st. Is he entitled to the credit of having been the first to discover and practically apply anæsthetics to the relief of pain in surgical operations?

There is no fact in human history better established, than that Dr. HORACE WELLS, of Hartford, Ct., commenced investigating the practical application of anæsthetics three years before Dr. MORTON dates his discovery with ether. He chose the nitrous oxide gas, and not only conceived the idea or principle of anæsthesia, but he clearly and successfully demonstrated its applicability, by administering it to patients with the effect of entirely preventing pain during the extraction of teeth. This is fully proved by the dentists and respectable citizens of Hartford. Having proved both its safety and efficiency in Hartford, Dr. WELLS went to Boston during the college term of 1843–4, was kindly received by Dr. J. C. WARREN, who informed the medical class that Dr. WELLS was in the college, that he claimed to have discovered an effectual method of relieving pain during surgical operations, spoke of the importance of the discovery, if it should prove true, and advised the students to see and hear what Dr. WELLS had to say.

Dr. WELLS lectured to the students and others on the subject, and performed some experiments, which excited among them much interest. During the same visit, he obtained the privilege of administering the gas to a patient in the Massachusetts General Hospital, for the purpose of having some operation performed, but through timidity, or some other cause, not enough was given to make the trial successful, and the attempt was reported a failure. Meeting with no encouragement for further trials, he left Boston, doubtless, feeling no little disappointment and chagrin, and returned to Hartford, where himself and other dentists of that city continued to administer nitrous oxide gas as an anæsthetic, with entire success, during the extraction of teeth. It is just here that the professional world has been most deceived in relation to Dr. WELLS. Because he left Boston in the winter of 1843–4, after a single unsuccessful trial of his anæsthetic, he has been persistently represented by Bostonians as having abandoned the effort to use or introduce anæsthetics in surgical practice. But this representation is fully proved to have been devoid of truth, Dr. WELLS never having abandoned the practical application of nitrous oxide gas to anæsthetic purposes to the day of his death. All these facts, in relation to Dr. WELLS, were perfectly familiar to Dr. MORTON, during the years 1844–5–6; and when the latter, in the fall of 1846, went to Dr. C. T. JACKSON, for a supply of nitrous oxide gas, it was with no other thought than to carry out the identical practice of Dr. WELLS. But on being told by JACKSON that ether would produce the same insensibility to pain, and was every way more convenient and manageable, he was induced to try it, and for the first time successfully during the extraction of a tooth, on the 30th of September, 1846; and on the 10th of October, 1846, he gave it with satisfactory results to a patient in the Massachusetts General Hospital, during an operation by Dr. J. C. WARREN. Such is the actual history of anæsthesia up to October, 1846. The entire *discovery* of Dr. MORTON consisted in substituting the use of ether, on the earnest and emphatic recommendation of Dr. C. T. JACKSON, for the nitrous oxide gas of Dr. WELLS. The act of Dr. MORTON bears the same relation to the preceding acts of WELLS, that the subsequent act of Dr. SIMPSON, in using chloroform, did to the use of ether by MORTON.

2d. Did Dr. MORTON, in his efforts to introduce the use of ether as an anæsthetic to the medical profession, meet with such opposition, such incredulity, such prejudice, and such misrepresentation that it required the sacrifice of his time, fortune, business, health, &c., to enforce so great a boon upon the attention of the world?

Let the facts contained in the first report on Surgery made to the American Medical Association, and published in the first volume of the transactions of that body answer.

Dr. MORTON'S first administration of the ether in the Massachusetts General Hospital, was on the 10th of October, 1846. On the 3d of November, less than thirty days thereafter, an account of it was read by Dr. HENRY J. BIGELOW, to the American Academy of Arts and Sciences, and on the 9th, to the Boston Society for Medical Improvement, and published by the same in the *Boston Medical and Surgical Journal* for Nov. 18th, 1846. The facts were also communicated to prominent members of the profession in London and Paris. Within six weeks, it had been fully and successfully tested in London, and in three months, it was in familiar use in the Hospitals of Paris, from which its use spread so rapidly over the whole of Europe, that in less than fifteen months Prof. SIMPSON had gathered and tabulated more than 300 cases of the larger amputations alone, in which ether or chloroform had been used. The use of these anæsthetics was adopted a little less rapidly in this country than in Europe, yet, from the report already alluded to, it appears that before the 1st of April, 1848, they were in almost daily use in every public hospital in the United States, and by all the distinguished surgeons North, South, East, and West. And not only this, but they had also been administered in "more than 2000 cases of obstetrics." Thus, within the incredibly short space of eighteen months, the use of anæsthetics had been fully introduced into the practice of surgery, obstetrics, and dentistry, throughout Europe and America, and that too without requiring Dr. MORTON to travel a mile, or expend a dollar, beyond the limits of Boston.

But why was the introduction of anæsthetics more slow in this country than in Europe? Let the able report on surgery, in the first volume of Transactions of the American Medical Association answer. (*See page* 181.) "This indifference to the discovery was probably owing to several causes. One of the *most prominent* of which was the *taint of charlatanism* which attached itself to its early history, and which created a prejudice against it, in some minds so strong as to prevent them from giving to the subject a full investigation. This feeling against secret and patented medicines, happily, prevails in the medical profession to a great extent, it is deep-rooted and sincere, and is based upon the highest considerations of public utility; if, in this instance, it was carried too far, the motive was, at least, just and honorable, and *the fault lies more with the discoverers* (MORTON and JACKSON,) *who attempted to conceal the nature of this new agent, under the name of* '*Compound Letheon*,' than with the profession." Thus it appears that, instead of opposition and prejudice on the part of the profession, the use of anæsthetics was adopted with a readiness and rapidity, both in Europe and America, unparalleled in the history of all previous remedial agents, and that whatever prejudice or neglect was exhibited towards them, was owing, mainly, to the unprofessional and quackish efforts of Dr. MORTON to conceal the nature of his anæsthetic under the false name of "Letheon," and to make all the world tributary to his pocket by a *patent right.* What disgusting misrepresentation then, for this same individual to be now perambulating the country, pretending to have sacrificed his business, his fortune, his health, and his all, in "*perfecting* and *introducing*" his great discovery to the world.

Pray what *perfecting* did it admit of, except to procure from the chemist a pure specimen of ether and strip its use of the cumbrous machinery, false names, and patents that Dr. MORTON had attempted to throw around it? The most diligent student of the history of anæsthetics will search in vain for any evidence that Dr. MORTON was ever required to abandon his legitimate professional business a single week, or spend a single dollar of his fortune beyond the cost of a few pounds of ether, in discovering, perfecting, and introducing his favorite anæsthetic to the profession and the world.

As we have already shown, his two first successful experiments were speedily described, endorsed, and heralded over both continents by eminent surgeons in Boston, and, with very few exceptions, accepted and acted upon with avidity by the profession at large. But we are confidently assured that Dr.

MORTON *did* sacrifice his business and his fortune, and undergo an immense amount of labor and anxiety, on account of his great discovery. And we have no reason to doubt the truth of the assertion.

This leads to our third and last question, namely:—For *what, and how, did he* make these great sacrifices?

To every impartial reader of history the answer is obvious. Dr. MORTON'S great primary animating idea was not the conferring of an inestimable boon upon the human race, by adding to our knowledge of the means of alleviating pain, but how to make that knowledge turn, with certainty, a pecuniary fortune into his own pockets. It was present with him before he had made his first successful demonstration, and prompted him to the dishonorable attempt to conceal the nature of his agent under the name of "Letheon." It was this that sent him in hot haste, not only to Washington for a *patent* covering all of this country, but across the ocean for one in England also. When he soon found that neither false names nor patents would enable him to control the coveted pecuniary profits, the same controlling idea constrained him to further abandon his legitimate business and squander whatever fortune was at his command in feeing eminent lawyers, and carrying out, through a protracted period, all the corrupting acts of a *lobbying* career, during the sessions of Congress, for the purpose of inducing the national legislature to endorse his pretensions, by an appropriation of a few hundred thousand dollars. All of which resulted in total failure, as was fitting and proper that it should.

If any of our readers think our remarks severe or captious, let them read the following preamble and resolutions, adopted almost unanimously, in a full meeting of the American Medical Association, held in the City of New York, in June, 1864:—

"*Whereas*, In the appropriation bill now pending in Congress, is a clause donating to Dr. W. T. G. Morton, of Boston, the sum of $200,000, as a recognition of his services in introducing sulphuric ether as an anæsthetic agent: and,

"*Whereas*, The said Dr. Morton, by suits brought against charitable medical institutions, for infringements of an alleged patent covering all anæsthetic agents, not claiming sulphuric ether only, put the state of anæsthesia, however produced, as his invention, *has, by this act, put himself beyond the pale of an honorable profession* and of true laborers in the cause of science and humanity; therefore,

"*Resolved*, That the American Medical Association enter their protest *against any* appropriation to Dr. Morton, on the ground of his unworthy conduct, also because of his unwarrantable assumption of a patentable right in anæsthesia; and, further, because private beneficence in Boston, New York, Philadelphia, and other places has already sufficiently rewarded him for any claim which he may justly urge.

"*Resolved*, That a copy of these resolutions be forwarded to the Chairman of the Committee of Ways and Means." (*See Transactions, vol xv., p.* 53.)

Also the following, which was adopted unanimously, by the American Dental Association, during its annual session, in Niagara, in July, 1864:—

"*Resolved*, By the American Dental Association, that to Horace Wells, of Hartford, Conn., now deceased, belongs the credit and honor of the introduction of anæsthetics in the United States of America; and we firmly protest against the injustice done to the truth and memory of Horace Wells in the effort made during a series of years, and especially at the last session of Congress, to award the credit to other person or persons."

Now, when Dr. MORTON will come before the profession and the world with a candid, truthful history of anæsthesia, giving to Dr. WELLS, Dr. JACKSON, and all others their just and proper credit, and claim for himself only just what he is fairly entitled to, namely, the credit of having by the suggestion of Dr. JACKSON, substituted for the inconvenient, unportable, and bulky nitrous oxide gas used by Dr. WELLS, the more convenient, portable, and manageable sulphuric ether, and so demonstrated its applicability as an anæsthetic as to, at once, command the attention of the scientific and professional world; and, at the same time, confess that all the business, money, and health he has heretofore sacrificed, has been in unsuccessful efforts, of a quackish and unprofessional character, to secure in some way the pecuniary profits of anæsthesia to himself, we will make a donation to the "Morton Testimonial," and invite all our friends to do the same. Until then, however, we must beg to be excused.

DEATH.—Dr. EZRA A. STEELE, who was associated with us, as assistant-editor, during the first one or two years of the publication of the MEDICAL EXAMINER, died at the residence of his uncle, in Marshall, Clark Co., Ill., on the 27th day of October last, aged about 30 years. We had known him intimately from his student days to the time of his death. He was a young man of more than ordinary talent, a physician of good judgment and skill, and a faithful friend. But consumption early marked him for a victim, and though he lingered long, yet, for the last four or five years of his life, he was almost wholly disqualified for active business. He had our warmest sympathies while living, and we extend the same to his large circle of relatives and friends in the hour of their bereavement.

---

ARMY SURGEONS RETURNED.—We learn, with pleasure, that our old and highly-esteemed friend, Dr. F. K. BAILEY, after serving ably and faithfully as an army surgeon, all through the late war for suppressing rebellion, has returned to his old field of civil practice, in Joliet, where we are sure he will be cordially welcomed by all his old friends and many new ones.

A few days since, we had the pleasure of meeting our former friend and faithful pupil, Dr. C. DUMREICHER, who had just graduated, with much credit to himself, in the spring of 1861. Only a month later, the first gun fired upon Fort Sumter awoke the nation to the realities of a gigantic war. Without waiting for a commission, our young and ardent friend joined almost the first mounted company raised in this State. But his medical attainments were soon called into requisition, and he was commissioned as assistant-surgeon. He served through the entire war with such faithfulness and satisfaction to the War Department, that he was promoted to the rank of Lieut.-Col. by brevet. He has now opened an office for practice in this city, and we hope he will receive all the patronage that his attainments and energy entitle him to.

---

SOMETHING WRONG.—We understand that since July last, the Surgeon-General has closed all the hospital records and public archives under his control, against the inspection of all persons outside of official positions. The reason unofficially alleged for this singular course is, that the Medical Bureau is preparing a work for publication, based upon these records, and that the Bureau is vain of being the instrument of the first publication of the facts.

Now, if these records were the private property of the Surgeon-General, procured at his own expense, there would be some justification for his turning his keys against all outside gleaners of facts and history. We suspect that Congress and the people have not authorized any such exclusiveness; and it remains to be seen whether they will approve the policy.

---

LONGEVITY OF CLASSES.—During the last Parliament of Gt. Britain, there have died 112 peers, the united ages of which amounted to 7583 years, or an average of 67 years each. The archbishops died at the average age of 80; the viscounts, at 74; the bishops, 73; the counts, 68; the marquises, 66; the dukes and barons, 64. The average age of the Scotch peers was 88; of the Irish, 63.

---

THE NEW ORLEANS SCHOOL OF MEDICINE was to open its regular annual term of lectures on the 13th of November, with many of its old faculty at their posts, and we doubt not but it will speedily attain to its previous prosperity and usefulness.

The Medical School at Nashville, we think, is also progressing in its active career, under the leadership of our old friend, Prof. W. K. BOWLING, who has been one of its faculty through all its days of former prosperity. We rejoice at all these evidences of returning enterprise in the profession of the Southern States.

---

### DEATHS.

TIMOTHY CHILDS, M.D., late Professor of Anatomy in the New York Medical College, died at Norwich, Conn., on the 3d inst., from an overdose sulphate morphia.

SIR WILLIAM HOOKER, an eminent English Botanist is dead.

Mortality for the Month of October.—The mortality report of municipal health department for the month of October has just been completed, and will be found below. The total number of deaths for the past month, as here reported, is 360, showing an increase of 90 over the corresponding month of last year. This increase is partly caused by the frequency of those diseases resulting from sudden and varying changes of climate, such as have prevailed during the month past; but it is chiefly owing to the remarkable growth of vegetation during the past season, owing to the lateness of the frosts, and which becoming rank, caused the prevalence of miasmatic and other vapors, inducing a state of atmosphere especially tending to produce disease:—

DISEASES.

| | | | |
|---|---|---|---|
| Accidents, | 7 | Inflammation of Lungs, | 4 |
| Apoplexy, | 1 | Intermittent Fever, | 1 |
| Brain Fever, | 3 | Lung Fever, | 1 |
| Billious Fever, | 7 | Liver Complaint, | 1 |
| Cholera Infantum, | 2 | Lung Disease, | 2 |
| Cholera Morbus, | 2 | Lockjaw, | 1 |
| Congestion of Brain, | 1 | Marasmus, | 1 |
| Congestion of Lungs, | 2 | Nervous Fever, | 7 |
| Cold, | 2 | Old Age, | 8 |
| Cancer in Head, | 1 | Paralysis, | 2 |
| Childbirth, | 6 | Rheumatism, | 1 |
| Congestion of Bronchial Tubes, | 1 | Stomach Disease, | 1 |
| Consumption, | 32 | Suicide, | 1 |
| Convulsions, | 6 | Stillborn, | 9 |
| Cramps, | 18 | Summer Complaint, | 14 |
| Croup, | 40 | Scrofula, | 1 |
| Decline, | 1 | Ship Fever, | 1 |
| Delirium Tremens, | 1 | Spasms, | 1 |
| Diarrhœa, | 11 | Sore Throat, | 7 |
| Diphtheria, | 36 | Scarlet Fever, | 7 |
| Dropsy, | 6 | Typhoid Fever, | 15 |
| Drowned, | 3 | Typhus, " | 2 |
| Dysentery, | 9 | Teething, | 18 |
| Erysipelas, | 1 | Tumor, | 2 |
| Green Canker, | 1 | Water on Brain, | 1 |
| Head Disease, | 1 | Whooping Cough, | 2 |
| Heart Disease, | 3 | Unknown, | 33 |
| Hemorrhage, | 1 | | |
| Inanition, | 1 | Total, | 360 |
| Inflammation of Bowels, | 10 | | |

Ages of the Deceased.—Under 5 years, 196; over 5 and under 10 years 33; over 10 and under 20, 13; over 20, and under 30, 23; over 30 and under 40, 37; over 40 and under 50, 18; over 50 and under 60, 12; over 60 and under 70, 10; over 70 and under 80, 10; over 100, 1; unknown, 7. Total, 360.

NATIVITIES.

| | | | | | |
|---|---|---|---|---|---|
| Chicago, | 193 | England, | 8 | Sweden, | 7 |
| Other States, | 73 | Germany, | 32 | Italy, | 1 |
| Bohemia, | 1 | Ireland, | 31 | Unknown, | 4 |
| Canada, | 3 | Norway, | 3 | | |
| Denmark, | 2 | Scotland, | 2 | Total, | 360 |

Revival of Medical Literature and Medical Schools in the South.—We have received an announcement, saying that the January number of a new journal, called the *Richmond Medical Journal*, would be issued early in December, to be edited and published by Drs. E. S. Gaillard and W. S. McChesney. It is to be published monthly, containing from 80 to 90 pages, with a subscription price of $5,00 per annum, payable in advance. We recognize in the name of Dr. Gaillard, an able and ready writer, and have no doubt but the journal, under his management, will be abundantly worthy of the patronage of the profession. We add it to our exchange list with much pleasure.

We have also received the following printed circular, which we insert in full:—

Sir:—I propose to edit and publish, in Memphis, Tennessee, *The Medical and Surgical Monthly*. The size of the journal will be not less than quarto; though I anticipate, from the encouragement of others and yourself, to give each month, to a large number of subscribers, not less than 64 pages of printed matter, interesting to mediciners, and important to patients. I will endeavor to give to the journal a character not unworthy either of the locality or the age of its publication.

Will you please to interest yourself in this matter, and return to my address, at an early day, the names of subscribers?

If this request is promptly and extensively responded to, the first number of the journal will be published in January, 1866.

Subscriptions will be received for six months, and one year: One year, Twelve Numbers, $6.00; One-half year, Six Numbers, $4.00. Very Respectfully,

FRANK A. RAMSEY, A.M., M.D.,

*Memphis*, *Tenn.*, *Oct.* 19*th*, 1865. *No.* 5 *Adams St.*

---

Military Surgery in France.—A work of paramount importance, modestly called a Report to the Medical Board of the Army, has lately been published by M. Chenu, a high authority in the Medical Department. It is entitled, "A Medico-Chirurgical Report on the Flying and Stationary Hospitals of the French Army, during the Crimean Campaign." It has no less than 800 quarto pages, and more than 100 tables and statistical computations. The author, says the *Gazette Medicale de Paris*, has been anxious to publish facts, irrespectively of every consideration; and we doubt not that the book will be eagerly sought by military surgeons in this country, as Mr. Longmore's is in France. M. Chenu is extremely minute in his classification, both as regards the field and hospitals; and the labor bestowed must have been immense. The comparative mortality of the French and British armies is thus stated:—

| | French Army. | British Army. |
|---|---|---|
| Effective men, | 309.268 | 97,864 |
| Deaths, | 95,615 | 22,182 |

The work is divided into numerous parts, including all the injuries and diseases which have been observed. Wounds from gunshot occupied much of the author's attention, and he dwells particularly on the fact that such wounds, owing to the peculiar firearms now used, are more serious, and more frequently met with than formerly. Herein, he quite agrees with Inspector Longmore, who states that the army of the Duke of Wellington, on the 16th, 17th, and 18th of June, 1815, including the battle of Waterloo, had 8000 wounded, whilst at Solferino, the Franco-Sardinian army had 16,000, and the Austrians 21,000. Hence, the necessity of increasing the surgical material and the medical officers in any future campaign. The author gives a vivid and extremely true picture of the perils and hardships of the non-combatant military surgeon. The following figures show the relative sacrifice of life among combatant officers and military surgeons:—In the Crimean campaign, of 5852 combatant officers of every rank, 779, or 17 per cent, were killed, or died from wounds received in the field, and 402, or 7·30 per cent, died of various diseases (these figures include the commissariat, chaplains, &c., &c.) The mean number of medical men was 450, of whom 82, or 18·22 per cent, died of various diseases. The typhus, at Constantinople, carried off 26 combatant officers, viz.: 0·17 per cent, whilst by the same scourge, 58 medical officers perished, viz.: 12·88 per cent. These figures speak for themselves.

---

Antagonistic Effects of Calabar Bean and Atropia.—Dr. Kleinwachter states (*Berliner Klinische Wochenscrift*, 1864,) that in the ophthalmic department of the hospital at Prague, last August, four boys engaged in cleaning the room, drank a portion of a solution of atropia, thinking that it contained spirits. Two of the boys either spat out or vomited the fluid, and exhibited no symptoms of poisoning; but the two others, who did not vomit, were distinctly poisoned—one, however, much more so than the other. The symptoms were those of poisoning by belladonna, and consisted of delirium, dilatation of the pupils, feeble pulse, and in one there was coma, alternating with furious delirium. Both the patients were taken to bed, one of them being restrained in a straight jacket, and cold lotions were placed on their heads. Dr. Kleinwachter happened, accidentally, to have with him a solution of the Calabar bean extract in glycerine, and, by way of experiment, he gave to the patient who was most affected, ten drops of the solution, (six grains of extract to one drachm of glycerine,) which, in about a quarter of an hour, produced violent vomiting. The pulse became stronger and quicker, rose to 75 and then to 80 in the minute, the temperature of the body fell, the delirium abated, the patient became more quiet, consciousness returned, urine was passed with some pain in the urethra, and the pupils became somewhat contracted. In the case of the other patient, who was less affected, some of the extract of Calabar bean was dropped into the eye, but without any good effect; for, on the next day, the symptoms were almost unchanged, while the patient who had taken the solution of the Calabar bean internally had almost completely recovered. The rapid and striking improvement in one of these cases appears manifestly to be attributable to the administration of the Calabar bean extract, for the patient who was not treated in the same manner showed no improvement for forty-eight hours.—*Brit. & For. Med. Chir. Rev., and Cincinnati Lancet & Obs.*

---

Entozoa in the Calf.—The recently-issued part of the "Proceedings of the Royal Society" contains a paper by Dr. Cobbold and Prof. Simonds, "On the production of the so-called 'acute cestode tuberculosis' by the administration of the proglottides of Tænia mediocanellata." The symptoms of the disease, which nearly caused the death of the calf, are fully detailed, and in the description of the *post mortem* examination all the muscles are named in which the parasites appeared. The authors conclude their paper as follows:—"From the number of young vesicles present in the minute portion of muscle removed by operation from the living animal, we had (in the pages of the *Lancet*) publicly announced our belief that we might ultimately find 30,000 cysticerci developed in this calf; but as the larvæ were subsequently found to be almost entirely confined to the superficial muscular layers, it turned out that our calculation was considerably beyond the mark. Nevertheless, from *post mortem* data, we estimate that there were between seven and eight thousand 'measles' present, and one of us counted 130 vesicles at the surface of a single muscle." The authors express their indebtedness to Dr. Greenhow, Dr. Anderson, and Mr. Brookhouse (Nottingham), for the specimens of tapeworm employed in this interesting experiment.—*Lancet.*

---

DIPHTHERIA.—*A Circular from Dr. Norton.*—Dr. O. D. Norton, of Cincinnati, was placed on a special committee by the American Medical Association to report on diphtheria. He has handed us the following list of queries, and any of our readers who have had opportunities for observing the disease, will confer a favor that will be appreciated and acknowledged, by corresponding with Dr. Norton:—

I. Has diphtheria occurred in your practice? If so, when did it first make its appearance? (Please state the year and the months in which it prevailed, and how many cases came under your observation.)

II. Did it occur as a sporadic, endemic, or epidemic disease?

III. Did the disease affect one class or age more particularly, and what were its general characteristics?

IV. What in your opinion are the general, and what the exciting cause or causes of the disease?

V. Do you consider diphtheria and scarlatina identical?

VI. Do you consider it communicable?

VII. What other diseases were especially prevalent at the time?

VIII. Do you know of any disease having affected animals during the occurrence of diphtheria in the community?

IX. Have you seen the diphtheritic membrane developed upon the cutaneous surface or upon wounds?

X. In what proportion of cases has the disease invaded the larynx? Also, the œsophagus?

XI. What have been the sequelæ?

XII. What has been the result of *post mortem* or microscopical examinations?

XIII. In what proportion of cases have you found albumen in the urine?

XIV. What has been the rate of mortality? and what the immediate cause of death?

XV. What was the general course of treatment pursued by you, and what particular remedial agents seemed to be most productive of good?—*From the Cincinnati Lancet & Obs.*

---

DRY GANGRENE OF THE HAND FROM INJECTIONS INTO AN ANEURISMAL SAC.—M. Chabrier has published in the *Mont-*

*pelier Medical*, the case of a man suffering from traumatic aneurism at the bend of the elbow. Compression having been tried in vain, an injection of five drops of solution of perchloride of iron into the sac was resorted to. The pulsations were thereby not arrested, and, as the patient seemed to have been but little inconvenienced by the injection of the coagulating fluid, a second operation of the same kind as the first was performed two days afterwards. The hand became immediately blanched, like a limb severed by amputation, and severe pain was complained of. The dry gangrene began at the tip of the fingers, and was in a fortnight so complete that the hand resembled a dried black claw. The line of demarcation had very early been observed about the wrist, and the hand was finally removed by a few touches of the scalpel. The aneurismal tumor had, in the meanwhile, become quite solid, and devoid of any pulsations. As the effect on the hand was so instantaneous, M. Chabrier thinks that the mischief was caused by embolism.

---

Appliances for Invalids.—At the recent Fair of the American Institute in New York, Mr. T. McElroy, of New York, had on exhibition a surgical operating table, which is so contrived as to put the patient in any position that may be required for the most difficult operation. The same inventor had an ingenious invalid bedstead.

Furman & Wells, of Addison, N. Y., exhibited O. P. Furman's patent invalid bedstead; and E. Marx, of New York city, had what he calls a patent patient elevator, for raising sick and disabled persons from their beds with ease and comfort, and placing them in any position nature or convenience may require.—*Med. & Surg. Rep.*

---

Singular Electrical Phenomena in the Human Body following Lightning Stroke.—M. Boudin recently sent a note to the French Academy of Sciences showing a powerful electric action in the bodies of persons recently struck by lightning, based on two observations which he related.

The first was the case of a man, who, June 30th, 1854, was killed by a stroke of lightning, near the Garden of Plants, at Paris, and whose body remained for some time exposed to a heavy rain. After the storm, two soldiers, wishing to raise the body, received each a violent shock at the moment when they touched it.

In the second case, two artillerists, charged with raising two electric telegraph posts which had been thrown down September

8th, 1858, by a storm at Zara, in Dalmatia, having, two hours after the storm was over, taken hold of the telegraph wire, felt at first two slight shocks, and then were suddenly thrown down. Both had their hands burnt; one of them, indeed, did not return to consciousness. The other, in attempting to raise himself, fell back again immediately on touching with his elbow one of his comrades, who had been drawn by his cries to his assistance. This last man, also thrown down in his turn, received various injuries of a nervous character, and his arm showed a burn on the skin where he had been touched.—*Boston Med. & Surg. Jour.*

---

Danger of Subcutaneous Injections.—Prof. Nassbaum, of Munich, has just published an interesting account of an accident which happened to himself. Suffering from neuralgia, he had injected morphia under his own skin more than 2000 times—sometimes to the extent of five grains of morphia in twenty-four hours. Two months ago, he injected two grains of acetate of morphia, dissolved in fifteen minims of water, and accidently sent it direct into a subcutaneous vein instead of into the cellular tissue. He gives a graphic account of his dangerous position for about two hours, after which the effect passed off. He has seen similar effects in a small degree in two of his patients, and the practical lessons are, that as it may be impossible to avoid veins at all times, and one may be punctured unawares, subcutaneous injection should always be done *very slowly*. The effects are so instantaneous that the syringe can be stopped at the first sign of danger, and some of the injected fluid, mixed with blood, may even be sucked out again by the syringe. It is very remarkable how the effects of the same dose of the same substance differ when injected directly into a vein and mixed with venous blood, and when they filter into the blood from the cellular tissue through the unbroken coats of the vessels.—*Med. Times and Gazette.*

---

Injection of Air into Foul Abscesses and Fistulæ.—Prof. Rozer states that there is no better means of treating an abscess, the contents of which have become foul and stinking, than by the frequent injection of air by means of an elastic catheter and syringe. The patient is to be instructed to repeat this injection sufficiently often to prevent the accumulation of pus; and this operation not only adds much to comfort by removing the nauseous smells, but also seems to greatly expedite the contraction and closure of the cavity of the abscess.

It is of especial benefit in the treatment of the fistulous openings remaining after emphysema, and attended with such a disgusting smell. This is speedily removed by aid of the injections, and the healing of the fistula promoted. This is often retarded for months, owing to the valvular condition of the track of the fistula preventing the free issue of the pus, which is secured by the daily passage of the catheter for the purpose of injecting the air.—*Archiv. der Heilkunde*, in *Medical Times and Gazette.*

---

Treatment of Choryza.—M. Luc, an Assistant-Surgeon in the French army, recommends the inhalation of tincture of iodine in nasal catarrh. "I inhaled tincture of iodine," says he, from a phial for one minute at a time, at intervals of about three minutes; the heat of my hand was sufficient to promote the evaporation of the iodine; the headache yielded first, sneezing became less frequent, the secretion less copious, and although the inhalation caused a burning sensation in the throat, I was cured at six o'clock P.M., of a cold which from nine A.M., to three P.M., had been sufficiently violent to compel me to use four pocket handkerchiefs."—*Cincinnati Lancet and Observer*, from the *Dublin Med. Press.*

M. Luc claims to have had equally good results in several other cases.

---

Records of the Medical Department.—Surgeon-General Barnes has sent a communication to the Secretary of War, setting forth the perilous condition of the records, etc., in the Medical Department, which is situated in a building in no way fire-proof, and by reason of its proximity to wooden buildings, liable at any moment to be burned up. Already the books and papers most valuable, in a scientific point of view, and to the families of deceased soldiers, have accumulated so that they occupy the entire story of a very large building. A proposition will be made in Congress immediately upon its organization, to construct fire-proof buildings for the State and War Departments, the latter to include suitable apartments for the Surgeon-General.—*Med. & Surg. Rep.*

---

A Fossil Man has been dug up in a bed of drift between Veyziat and Oyonnax, near Nantua, (Ain). The body was found in an inverted posture, but the bones did not adhere together, but were brought out one by one. The matter will probably be brought before the the Academy of Sciences.—*Abeille du Bugey.*

LONGEVITY IN ENGLAND AND WALES.—The returns of mortality for England in the year 1863, just completed, record the death of 213 men and 430 women ninety-five years old or upwards when they died. Twenty-one men had reached 100 or upwards, and one at Chelsea was 109. Sixty-two women had also completed a century of life or more, and one at Liverpool was 112 years old.

PROF. MALGAIGNE.—We regret to state that the health of this eminent surgeon is far from improving. He has just resigned his surgical chair at the Faculty of Medicine at Paris.

A PRIZE ON THE VACCINO-SYPHILITIC QUESTION.—The Medico-Chirurgical Society of Bologna (Italy) offers for 1867, a prize of £20 on the following question: Determine by facts whether the vaccine virus may or may not transmit syphillis. Another prize (£40) is offered on the subjoined subject: Make known, and give its proper value to, the share which Italians have had in the advancement of surgery in the nineteenth century.—*London Lancet.*

LONDON LANCET.—The December number of this standard monthly of English medical periodical literature, is promptly on our table, with its usual rich variety of practical and interesting reading matter.

